# Orofacial findings and orthodontic treatment conditions in patients with down syndrome – a retrospective investigation

**DOI:** 10.1186/s13005-023-00362-5

**Published:** 2023-05-06

**Authors:** Stephan Christian Möhlhenrich, Peter Schmidt, Sachin Chhatwani, Kristian Kniha, Alan Tsipkis, Joachim Jackowski, Andreas G. Schulte, Gholamreza Danesh

**Affiliations:** 1 Department of Orthodontics, University of Witten/Herdecke, Alfred-Herrhausen Str. 45, 58455 Witten, Germany; 2 Department for Special Care Dentistry, University of Witten/Herdecke, Alfred-Herrhausen Str. 45, 58455 Witten, Germany; 3 Department of Oral and Maxillofacial Surgery, University Hospital of Aachen, Pauwelsstraße 30, 52074 Aachen, Germany; 4 Department of Oral Surgery, University of Witten/Herdecke, Alfred-Herrhausen Str. 45, 58455 Witten, Germany

**Keywords:** Down syndrome, Trisomy 21, Orthodontic therapy, Orthodontic treatment need, Malocclusion

## Abstract

**Introduction:**

The most common chromosomal anomaly is Down syndrome/Trisomy 21, which can be associated with varying degrees of intellectual disability and physical malformation. Specific orofacial characteristics regarding orthodontic treatment options and features are described on the basis of a patient collective from the Witten/Herdecke University, Germany.

**Methods:**

Data of 20 patients (14 boys and 6 girls, mean age: 11.69 ± 3.94 years) who underwent orthodontic treatment between July 2011 and May 2022 were analyzed. Baseline skeletal and dental conditions were assessed, as well as the presence of hypodontia, displacements, and treatment-related root resorptions. The treatment need was evaluated based on the main findings according to the German KIG classification. In addition, treatment success was determined in relation to patient compliance.

**Results:**

The patient group was characterized predominantly by a class III relationship (ΔANB: −2.07 ± 3.90°; ΔWITS: −3.91 ± 4.33 mm) and a brachyfacial cranial configuration (ΔML-NL: −4.38 ± 7.05°, ΔArGoMe: − 8.45 ± 10.06°). The transversal discrepancy of the dental arch width from maxilla to mandible was −0.91 ± 3.44 mm anteriorly and −4.4 ± 4.12 mm posteriorly. Considering the orthodontic indication groups, the most frequent initial finding and treatment indication represented hypodontia (85%), followed by frontal (75%) and unilateral lateral (35%) crossbite. In 55% of the cases, the teeth had a regular shape, but in 35% a generalized and in 15% an isolated hypoplasia. Only 25% of the patients could be treated with a fixed multiband appliance due to sufficient cooperation. In each of these patients, varying degrees of root resorptions were detected during treatment, and 45% of all treatments had to be terminated prematurely due to a lack of cooperation by patients or parents.

**Conclusion:**

The extent of dental and skeletal malformations and the high rate of findings requiring treatment in patients with Down syndrome represent a significant indication for orthodontic therapy, which can be well illustrated by the KIG classification. However, this is in contrast to the eventually increased risk of root resorption, with significantly reduced patient cooperation. A compromised treatment outcome and process must be expected. Consequently, the orthodontic treatment must be simple and realistic to achieve fast and therapeutically satisfactory treatment result.

## Introduction

Down syndrome (DS) is the most common genetic disorder associated with intellectual disability and is characterized by a variety of other clinical findings. The incidence is approximately 1 in 800 births in the United States, while for Europe, between 2011–2015, 8031 annual live births of children with DS and a live birth prevalence of 10.1 per 10,000 live births was estimated. Without elective terminations, LB prevalence would have been around 21.7 per 10,000 live births, or 17,331 births annually. [1, 2]. The syndrome was first described in 1866 by the English physician John Langdon Down [3]. More than 90 years later, the chromosomal cause was described, and the condition was named Down syndrome [4, 5]. This genetic abnormality is caused by the presence of a complete or partial third copy of chromosome 21 and is typically associated with characteristic facial features, physical growth delays, and mild to moderate intellectual disability [6]. Characteristics of DS are considerable phenotypic differences between individual patients. These include, among others, learning disability with varying degrees of expression, however often combined with high skills regarding social intelligence, empathy, and social integration [7].

These patients are often associated with other conditions and medical problems of different extents. These include abnormal eye alignment related to squinting as well as long- or short-sightedness, hearing problems, and ear infections. Hearing problems are especially important concerning a child’s development and must be recognized at an early stage [8]. In addition to these general health problems, individuals with disabilities or syndrome related conditions have shown different findings with regard to their oral health status [9–12]. In this context, a generally lower caries prevalence is reported for this group of individuals; in contrast to this, a greatly increased risk of developing periodontitis is described [13–15].

Specifics with regard to maxillofacial development and the possible need for orthodontic treatment are known in patients with DS [16, 17]. These include, among others, delayed tooth eruption, congenitally missing teeth, tooth size discrepancy, impacted or transposed teeth, tongue thrust and protrusive tongue posture, open bite, and maxillary anteroposterior or transverse hypoplasia [1, 18]. The typical skeletal anomaly is characterized by an Angle Class III tendency (54%), posterior crossbites (65%), and a frontal open bite tendency [1].

Even though malocclusion is not a disease and is often associated with a higher degree of subjectivity and distorted with insights about the treatment need, general reasons for orthodontic treatment are to improve oral or dental health, chewing function, and dental or facial esthetics [19, 20]. Recently, Alkawari investigated the need for orthodontic treatment in patients with DS using the Index of Orthodontic Treatment Need (IOTN) in a cross-sectional study [8]. The results showed that a high percentage of children needed orthodontic treatment (81.9%). Of these, 59.1% presented with Angle’s class-III malocclusion, while 36.4% showed class I occlusion. Concerning relationships between the IOTN and gender, no statistically significant differences were found [8]. The author concluded that a higher percentage of patients with DS had very severe malocclusion, which is why treatment can be considered obligatory in general [8]. Furthermore, it was reported that more than three-fourths of the children had already visited a dental clinic, but 30.4% of the children’s mothers declared that no orthodontist had been consulted so far.

In 2002, a system of orthodontic indication groups [Kieferorthopädische Indikationsgruppen (KIG)] was introduced in Germany [21] (Table 1). This system is a derivative of the IOTN and is used to regulate access to orthodontic treatment in the German public health insurance system, taking into account the malocclusion and the corresponding severity grade [21–23]. Therefore, craniofacial anomalies such as cleft palate and syndrome-related oral manifestations represent the highest degree of severity. Accordingly, DS was assigned to this highest indication group. However, in patients with DS, the malocclusions corresponding to this classification have not been described so far.

**Table 1:** Classification of orthodontic treatment need using German orthodontic indication groups (KIG). A severity grade score more than or equal to 3 is the cut-off for orthodontic treatment for children (aged less than 18 years) in the public health insurance system

Malocclusion		Severity grade				
		1	2	3	4	5
A	Craniofacial Anomalies					Cleft palate and syndromes
U	Missing teeth				Agenesis or loss	
S	Disturbance in tooth eruption				Impaction	Displacement
D	Sagittal discrepancy increased overjet	< 3 mm	3–6 mm		> 6–9 mm	> 9 mm
M	Sagittal discrepancy negative overjet				0–3 mm	> 3 mm
O	Vertical discrepancy open bite	< 1 mm	> 1–2 mm	> 2–4 mm	> 4 mm, habitually open	> 4 mm, skeletally open
T	Vertical discrepancy deep bite	> 1–3 mm	> 3 mm, with / without mucosal contact	> 3 mm, with traumatic mucosal impingement		
B	Transverse discrepancy				Scissors bite	
K	Transverse discrepancy crossbite		Buccolingually cusp-to-cusp relation	Bilateral crossbite	Unilateral crossbite	
E	Contact point displacement	< 1 mm	> 1–3 mm	> 3–5 mm	> 5 mm	
P	Space deficiency		< 3 mm	> 3–4 mm	> 4 mm	

**Table 2A:** Cephalometry initial findings with regard to the skelettal sagittal and vertical craniofacial configuration of a study group of 20 patients diagnosed Down Syndrome

Patient	Gender	Sagittal cranial configuration			Vertical cranial configuration					Anterior tooth angulation	
		ΔWITS (NR: 0 ± 1 mm)	Δ ANB (NR: 2.0 ± 2°)	Overall Outcome (class)	Δ ML-NSL (NR: 32 ± 2°)	Δ NL-NSL (NR: 8.5 ± 2°)	Δ ML-NL (NR: 23 ± 3°)	Δ ArGoMe (NR:128 ± 7°)	Overall Outcome	Δ UP1-NSL (NR: 102 ± 2°)	Δ LO1-ML (NR: 90 ± 3°)
1	m	0	-0.8	I	-1.7	3.4	-5.1	-7.1	brachyfacial	9.1	4.3
2	m	-2.9	-7.7	III	-25.4	-7.2	-18.2	-30.8	brachyfacial	26.6	17.9
3	f	-6.7	-4.5	III	-1.5	2.5	-3.9	-9.3	brachyfacial	21.8	8.5
4	f	-8.1	-6.5	III	-6.5	-0.5	-6.5	0.1	brachyfacial	10.5	-15.5
5	f	-1.8	-3.4	III	-7.1	-1.8	-9.8	-7.2	brachyfacial	12.6	6.3
6	m	3.4	6.6	II	9.2	-0.5	9.7	4.9	dolichofacial	-1.2	-3.1
7	m	-7.2	-0.3	III	-2.6	-4.1	1.5	-9.5	brachyfacial	18.6	10.8
8	m	-4.4	-5.4	III	-7.5	-1.2	-6.3	-15.5	brachyfacial	13.7	-2.7
9	m	-3	-3.4	III	-3.6	-0.7	-2.9	-0.4	dolichofacial	-15.8	-6.5
10	m	-4.1	-2.2	III	-5.7	-1.8	-3.9	-17	brachyfacial	-0.7	-13.9
11	m	-6.4	-2.3	III	-2.4	-0.8	-1.6	-5.4	brachyfacial	2.6	4.4
12	f	-3.2	-0.3	III	-8.3	-5.7	-2.6	-16.6	brachyfacial	18.8	15.6
13	m	0.8	2.6	I	1.3	-0.4	-17.6	-9.8	brachyfacial	5.4	2.7
14	f	-14.9	-6.3	III	-7.4	0.4	-7.8	0	brachyfacial	15	-5
15	m	1.5	2.7	I	8.9	-0.8	9.7	5.2	dolichofacial	1.1	-1.3
16	m	0.2	-1.6	I	-0.8	0.6	-1.4	-21.9	brachyfacial	-3.6	8.1
17	f	-6.2	-7.9	III	-10.2	-0.9	-9.3	-14.5	brachyfacial	16.2	5.2
18	m	-9.9	-4.1	III	8.3	9.8	-1.6	-5.4	brachyfacial	-1.6	-18.9
19	m	-4	0.2	III	-0.7	-0.3	-0.4	-9.5	brachyfacial	-4	10
20	m	-1.2	3.2	III	-11.2	-1.7	-9.6	-18.4	brachyfacial	17.3	26.1
Mean, SD		-3.91 ± 4.33	-2.07 ± 3.90		-3.75 ± 7.83	-0.59 ± 3.42	-4.38 ± 7.05	-8.45 ± 10.06		8.12 ± 10.78	2.65 ± 11.35

NR: Normal range, Δ: Difference to norm, *: Deviation from the norm

Trisomy 21 causes muscular hypotonia which, if left untreated, can result later in a high need for orthodontic treatment. If oral hygiene deficits are not compensated, this can lead to a comparatively poorer oral health status. The question arises whether a classical orthodontic treatment is possible in adolescence and adulthood in persons with DS and what influence it can have on maxillofacial and oral health condition.

Therefore, the present retrospective investigation was performed to describe the initial orthodontic findings in patients with DS according to the KIG system and to analyze the realizable extent of orthodontic treatment concerning their compliance. In addition, unexpected specific events during the treatment should be identified.

## Materials and methods

Ethical approval to conduct this retrospective study was obtained from the institutional review board of the Ethics Commission of the University of Witten/Herdecke, Germany (No. S-194/2022).

The present retrospective investigation was based on patients with DS who visited the interdisciplinary consultation service of the Department of Orthodontics as well as Special Care Dentistry between July 2011 and May 2022. Early infant treatment according to the Castillo-Morales protocol was not included [24]. In order to be included in the study, potential patients had to be sufficiently compliant to generate orthodontic diagnostic documentation. This included alginate impressions of the upper and lower jaw as well as corresponding bite registration for situation models, the preparation of a panoramic radiograph and a lateral cephalogram, and intraoral and extraoral photographs. Furthermore, it had to be recognizable from the treatment documents which devices were intended and if they were actually used. In addition, the course of treatment had to be evident and whether there were any restrictions during treatment. These included inadequate oral hygiene, compliance with treatment appointments, and the handling of the appliance (wearing time, care, damage, loss). From the 71 initial consultations, these documents could be used from 20 individuals. Accordingly, in 51 patients no complete initial orthodontic examination could be performed and consequently no therapy started. The reasons were lack of need treatment, insufficient patient compliance with regard to the diagnostic assessment as well as inadequate oral hygiene despite repeated instructions and checks.

**Table 2B:** Clinical initial situation and orthodontic findings with regard to a study group of 20 patients diagnosed Down Syndrome

Patient	Gender	Initial findings according to KIG			Anterior teeth		Transversal dental arch difference (Δ)		Tooth anomalies			
		1°	2°	3°	OJ	OB	anterior	posterior	Number of missing teeth	Hypodontia	Dental hyoplasia	Displacement
1	m	U4	K4	P3	2.5	2.5	5	1	1	32	none	none
2	m	S5	U4	M4	3	1	-3.6	-2.1	6	12, 35, 31, 41, 42, 45	generalized	15
3	f	U4	M4	K4	-2.5	-1.8	0	-6	2	25, 42	generalized	none
4	f	S5	U4	M4	0	1	2.3	-5	10	15, 12, 22, 25, 35, 31, 41, 45	generalized	none
5	f	S5	U4	B4	0	-1.8	4.2	-2.3	2	12, 22	none	15, 25
6	m	U4	M4	K3	3	0	-8.1	-10.1	2	17, 27	none	none
7	m	U4	M4	K3	-1	-2	-7	-13	1	25	generalized	none
8	m	U4	M4	K3	-3	-6	0.7	-7.1	3	12, 22	generalized	14, 44
9	m	U4	S4	M4	-3	-2	1.5	5.2	1	45	none	none
10	m	M4	P4	E3	-1	0	1.5	-5.5	0	0	none	none
11	m	M4	K4	P3	-2	2	-3.7	-4.8	0	0	none	none
12	f	S5	U4	K4	3.5	2.5	0.1	-2.6	6	15, 12, 22, 25, 35, 45	generalized	14, 24
13	m	U4	P4	E3	6.2	3.8	-2.9	-3.8	1	35	isolated (12, 22)	none
14	f	U4	M4	K3	-1	1	-1.1	-4.9	2	35, 45	none	none
15	m	U4	O4	K4	2.5	-4.1	-1.3	-3	4	12, 22, 32, 31, 41	isolated (44, 45)	none
16	m	U4	M4	K4	0	0	-4.5	-7.7	1	12	none	none
17	f	M4	E3	P3	-2	3	0.3	-4	0	0	generalized	none
18	m	U4	M4	K4	-0.5	0	2.5	-2.5	1	31	none	none
19	m	U4	S4	M4	-2	0	-1.5	-9	1	35	none	none
20	m	U4	M4	K3	0	0.5	-2.5	-0.6	2	35, 45	none	none
Mean, SD					0.14 ± 2.52	-0.02 ± 2.41	-0.91 ± 3.44	-4.39 ± 4.01	2.3 ± 2.5			

OJ: Overjet. OB: Overbite. Δ: Upper – Lower dental arch, SD: Standard deviation

### Cephalometric analysis

The skeletal analysis is based on established cephalometric measurement distances and angles to characterize the sagittal and vertical cranial configuration [25, 26]:

- Sagittal relationships: Deviation (Δ) from ANB (sagittal interbase angle, °) and Wits appraisal (mm).
- Vertical relationships: Deviation (Δ) from NL/NSL (maxillary inclination, °), ML/NSL (mandibular inclination, °), ML/NL (vertical interbasal relationship, °), and the gonial angle (ArGoMe, °).
- Anterior tooth angulations: Deviation (Δ) from UP1-NSL (upper incisor inclination, °) and LO1-ML (lower incisor inclination, °).

### Clinical analysis

The description of the clinical findings was carried out according to the KIG classifications (Table 1) and based on the clinical inspection of the oral cavity, the evaluation of the panoramic radiograph, and subsequently generated situation models.

- *Main findings*: According to the KIG classification, the three most severe findings (primary, secondary, and tertiary) in addition to the obligatory severity grade A5 (presence of the diagnoses of DS) were determined from the initial diagnostic records.
- *Tooth anomalies*: Hypodontia, tooth retention, and displacement concerning the type and number were determined by panoramic radiograph and situation model analysis. In addition, root resorptions that occurred during treatment were recorded.
- *Model analysis – linear measurements*: Anterior tooth relation: Overjet (mm), overbite (mm); transversal dental arch relations. The width of the maxillary dental arch was measured between the central fissure of the first premolars (anterior, P1_Up_) and between the central fissure of the first molars (posterior, M1_Up_). In addition, the width of the mandibular dental arch was measured between the distal ridges of the first premolars (anterior, P1_Low_) and the distobuccal cusp tips of the first molars (posterior, M1_Low_). The relation between maxillary and mandibular arch widths was determined as the difference (Δ) between the respective distances: anterior = P1_Up_ − P1_Low_; posterior = M1_Up_ − M1_Low_.

### Treatment analysis

The progress of treatment was taken retrospectively from the patient’s medical record and based on the determination of the treatment duration (start and ending of the active therapy), the need for adjunctive logopedics, the achievement of the treatment goal, and, in the case of non-achievement, the reasons for this.

## Results

**Fig. 1:**
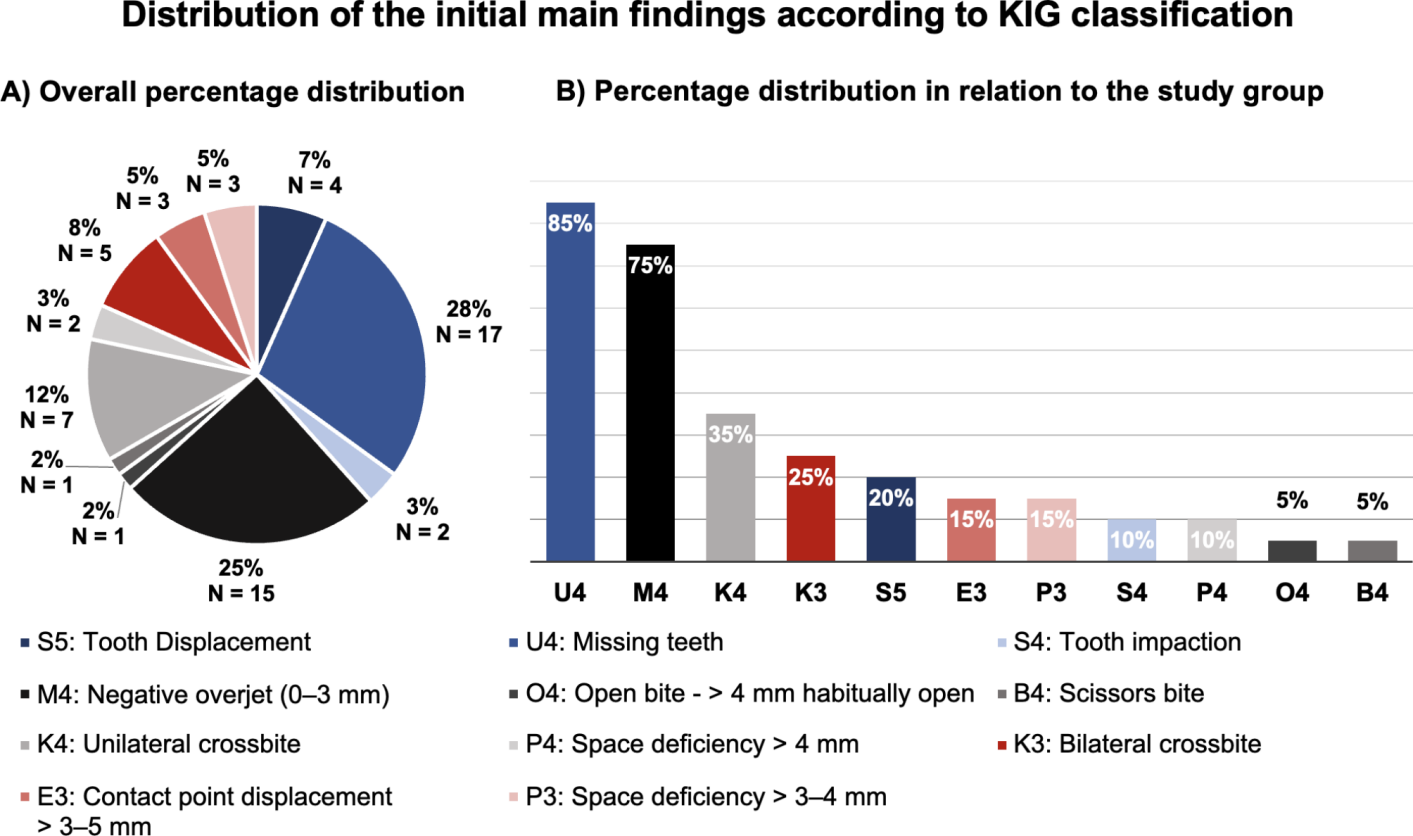
**A)** Percentage distribution of all orthodontic findings (100%) within the study group. **B)** Percentage distribution of individual orthodontic findings in relation to the study group (100%)

The studied collective was based on 20 persons – 6 female and 14 male patients. The mean age at the time of the initial consultation was 11.69 ± 3.94 years. Figure 1 illustrates the percentage distribution of the main findings. With regard to the clinical initial conditions, the most frequent finding of all diagnoses according to the KIG classification was missing teeth, U4 (85%, N = 17), with an average number of missing teeth per patient of about 2.3 ± 2.5. This was followed by negative overjet, M4 (25%, N = 15), and unilateral crossbite, K4 (12%, N = 7). This means for the study group an incidence of hypodontia of 85%, and 75% for the anterior and 35% for the unilateral lateral crossbite.

The initial clinical and radiological situation of all 20 patients is shown in Table 2 A and 2B. The anterior teeth were clinically characterized by a tendency to an edge-to-edge bite due to an average overjet of 0.14 ± 2.52 mm and an overbite of −0.02 ± 2.41 mm. Concerning the transversal dental arch differences, these indicated a crossbite mainly in the posterior arch segment (anterior: −0.91 ± 3.44 mm, posterior: −4.39 ± 4.01 mm). Furthermore, regarding the natural material of the teeth, 45% of patients (N = 9) demonstrated isolated or generalized dental hypoplasia. With regard to missing teeth, the right lateral incisor and the left second premolar were most frequently affected, both 7 times. Displacements were found in 5 patients (25%) and always affected the maxillary premolars. According to the WITS and ANB, skeletal class III was found for the majority of the study group (ΔWITS: −3.91 ± 4.33 mm; ΔANB: −2.07 ± 3.90°). Thus, 15 patients showed class III (75%), 4 patients class I (20%), and 1 patient class II (5%). With regard to the vertical dimension on average, a dolichofacial skull configuration (ΔML-NL: −4.38 ± 7.05°, ΔArGoMe: −8.45 ± 10.06°) was found. Of the patients, 85% (N = 17) showed a brachyfacial configuration and 15% (N = 15) showed a dolichofacial configuration. No mesofacial types were identified. Regarding the inclination of the anterior teeth, there was a significant proclination compared to the normal range for the maxillary anterior teeth, whereas the mandibles were orthograde on average (ΔOK1-NSL: 8.12 ± 10.78°; ΔUK1–ML: 2.65 ± 11.35°).

**Fig. 2:**
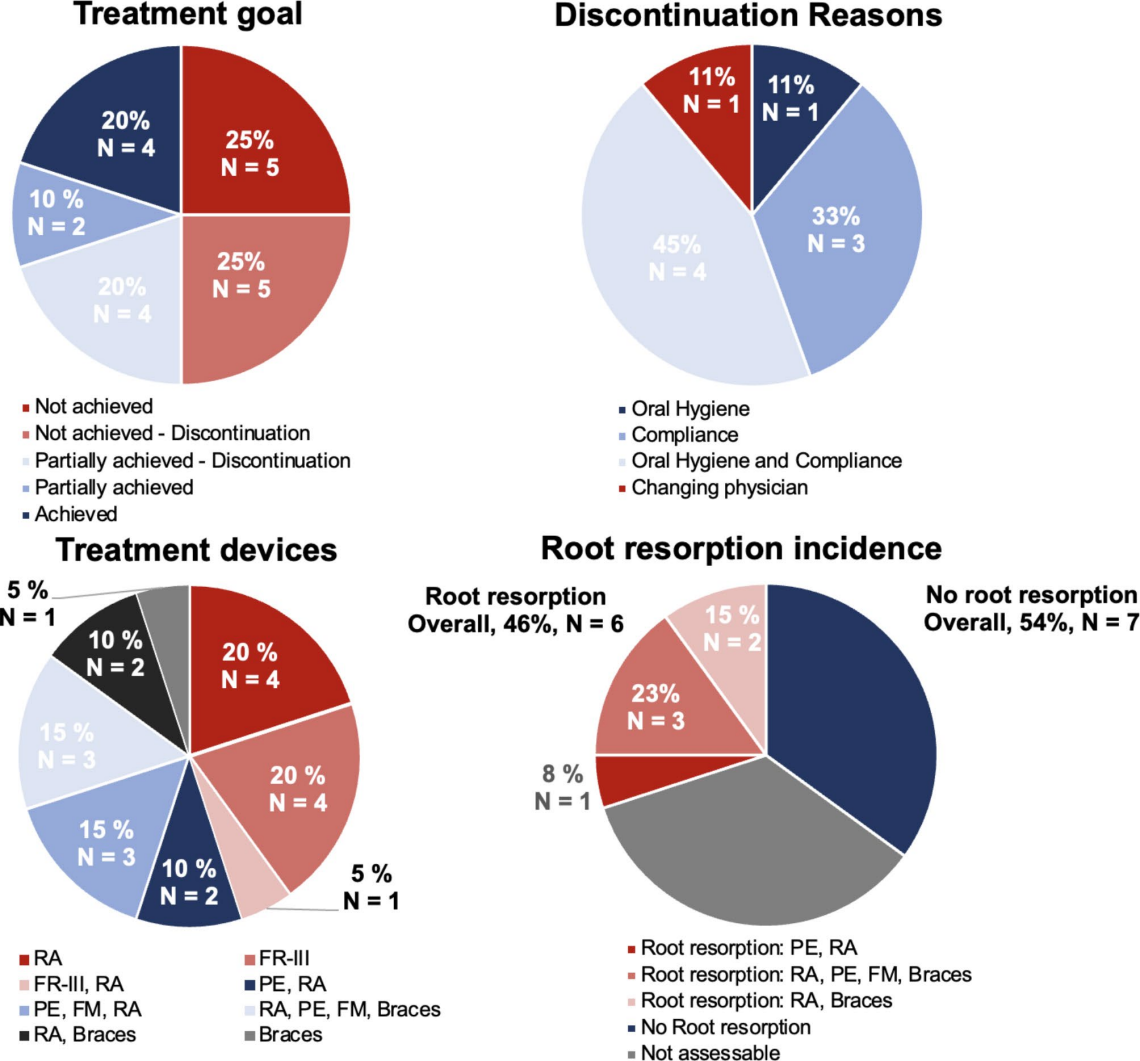
Treatment-related percentage distributions with regard to treatment goals, reasons for discontinuation, used treatment devices, and incidence of treatment-related root resorptions in 20 patients with a diagnosis of Down syndrome

**Fig. 3:**
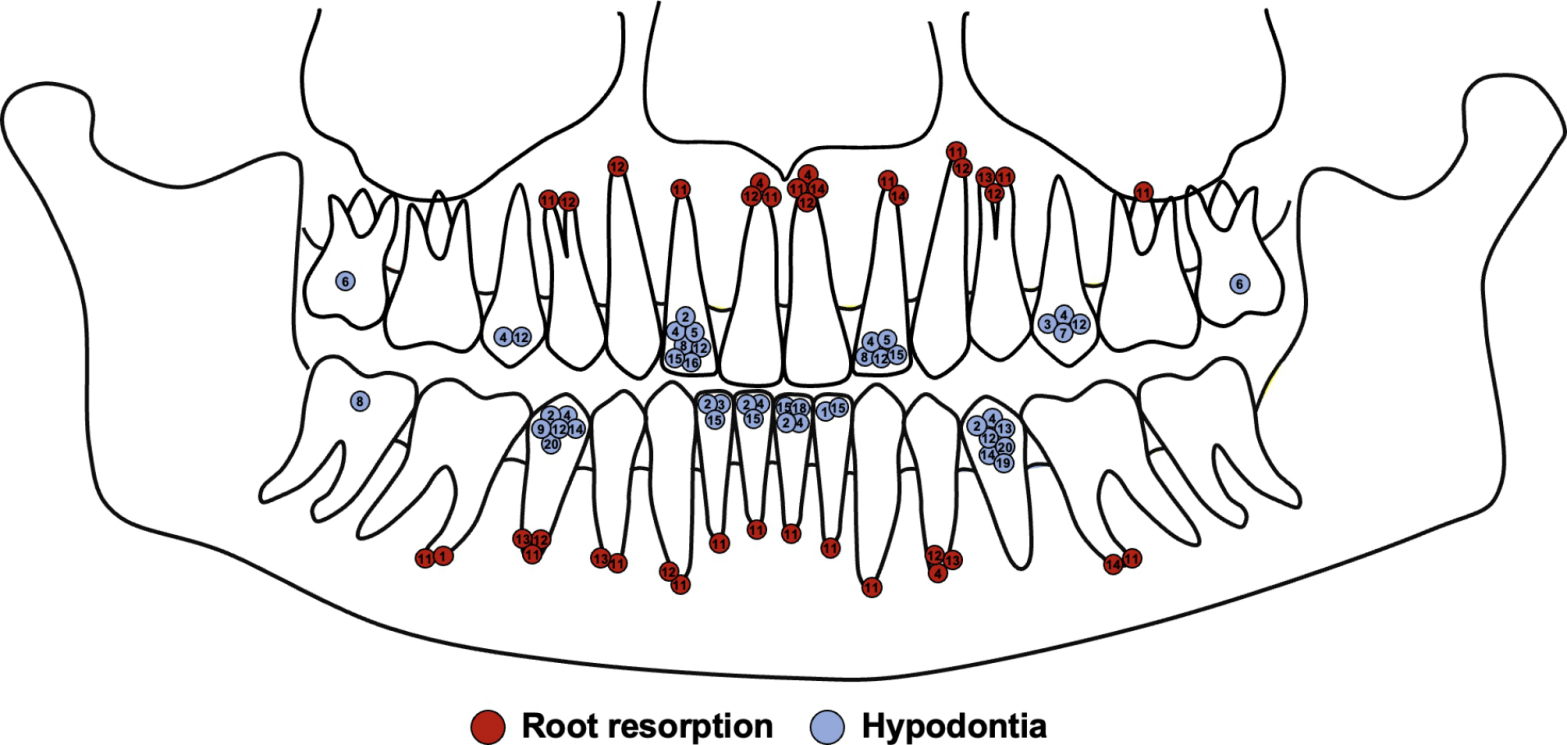
Systematic illustration of the affected teeth concerning the occurrence of root resorptions and hypodontia in the study group

Information about the treatment concerning patients’ age, main findings, treatment time, success rate, and associated reasons for treatment failure or discontinuation is presented in Table 3. In addition, Fig. 2 demonstrates the percentage distribution of the treatment success, reasons for discontinuation, the kind of treatment devices used, and the incidence of root resorption during the treatment. The orthodontic treatment started on average at the age of 13.26 ± 3.78 years and lasted, on average of 3.20 ± 2.19 years. In 65% (N = 13) of the treatment cases, supportive logopedic treatment was necessary. In general, the orthodontic and orofacial treatment was based on three different basic treatment concepts: (1) removable orthodontic appliance (RA) and/or Frankel functional regulator III (FR-3) in 45% of the treatments (N = 9), (2) palatal expander (PE) (with facemask (FM)) with/without removable appliance in 40% of the treatments (N = 8), and (3) fixed appliance (braces with/without palatal expander with facemask) in 15% of the patients (N = 3). Unfortunately, 45% of the treatments (N = 10) did not result in a successful outcome. Partial success was seen in 30% of all treatments (N = 6), and the treatment goal was achieved in only 25% of the treatments (N = 4). In this context, reasons for treatment discontinuation and failure were missing compliance (33%, N = 3), oral hygiene (11%, N = 1), or a combination of both (33%, N = 3). In 13 patients, radiological follow-ups could be performed using panoramic radiographs during or after the treatment. Varying degrees of treatment associated root resorptions were detected in 6 patients (46%). All of them were treated with a kind of fixed appliance (palatal expander and/or braces). An illustrated overview of the affected teeth with regard to treatment-related root resorption can be found in Fig. 3.

**Table 3:** Treatment-specific results regarding the extent and success of the planned therapy

Patient	Age during Treatment			Treatment					
	First Visit	Start	End/ Last visit	Duration	Intended Devices	Logopedics	Treatment goal achieved	Main Reasons	Final Root Resorption
1	12.1	15.7	18.1	2.4	PE, RA	Yes	Discontinuation, Partially achieved	Oral hygiene, Compliance	1
2	13.2	13.8	16.3	2.5	FR-3	Yes	Not achieved	Compliance	None
3	11.4	11.8	14.9	3.1	FR-3	Yes	Not achieved	Compliance	None
4	10.7	10.8	18	7.2	RA, PE, FM, Braces	Yes	Achieved	None	3
5	16	17.2	18.7	1.5	RA	Yes	Discontinuation, Not achieved	Oral hygiene, Compliance	None
6	10.8	11.8	14.2	2.4	PE, RA	No	Discontinuation, Partially achieved	Oral hygiene, Compliance	NA
7	9.7	10.7	14.3	3.6	FR-3, RA	No	Not achieved	Compliance	None
8	11.5	11.9	15.3	3.4	FR-3	Yes	Discontinuation, Partially achieved	Change of practice	NA
9	9.3	9.9	10.9	1.0	FR-3	No	Discontinuation, Not achieved	Compliance	NA
10	9.9	10.6	12.4	1.8	PE, FM, RA	No	Not achieved	Compliance	None
11	10.2	11.1	16.6	5.5	RA, PE, FM, Braces	Yes	Achieved	None	18
12	24.3	25	30.8	5.8	RA, Braces	Yes	Achieved	None	9
13	6.3	9.2	15.7	6.5	RA, Braces	No	Achieved	None	4
14	11.3	15	22.5	7.5	RA, PE, FM, Braces	No	Partially achieved	Compliance	3
15	8.7	10.1	10.9	0.8	RA	Yes	Discontinuation, Not achieved	Compliance	NA
16	7.7	9.8	12.9	3.1	PE, FM, RA	Yes	Partially achieved	Changing physician	None
17	10.9	12.8	15.1	2.3	PE, FM, RA	Yes	Not achieved	Compliance	None
18	17	17.5	18	0.5	Braces	No	Discontinuation, Not achieved	Oral hygiene	NA
19	14.1	16.3	16.7	0.4	RA	Yes	Discontinuation, Not achieved	Compliance	NA
20	8.7	14.2	16.9	2.7	RA	Yes	Discontinuation, Partially achieved	Oral hygiene, Compliance	NA
Mean, SD	11.69 ± 3.94	13.26 ± 3.78	16.41 ± 4.37	3.2 ± 2.20					

NA: Not assessable. PE: Palatal expander. RA: Removable Appliance. FR-3: Functional regulator type 3. FM: Facemask

## Discussion

About 83% of individuals with DS have severe malocclusions [27]. Therefore, the orthodontic treatment need in this population is objectively very high and consequently undisputed [28]. Many studies address treatment needs and corresponding orofacial findings. It is clear from these that there are specific malocclusion trait components for these subjects. However, little is reported on actual treatability. This could be significantly reduced concerning the fact that patients with DS often have varying degrees of cognitive impairments. Therefore, the aim of the present retrospective analysis was, on the one hand, to determine the initial therapeutic conditions with regard to the craniofacial development of patients with DS and the associated need for treatment and, on the other hand, to examine the extent to which orthodontic treatment was possible.

In the present study, the data of patients with DS were retrospectively analyzed from those in whom a treatment indication was determined. The mean age of these patients was 11.69 ± 3.94 years and is comparable to general orthodontic patients in university orthodontic care, which is about 12.1 ± 3.5 years [29]. This can be explained by the fact that only patients with a proven need for orthodontic treatment according to the KIG classification were included in the present retrospective analysis. Unfortunately, no information was given about patients with DS who had presented with no orthodontic treatment need. Therefore, the initial need for orthodontic treatment in this group could not be determined. Instead, the severity of malocclusion was evaluated in the present investigation based on the German KIG classification. For this purpose, the initial clinical and radiological situation was evaluated, and the orthodontic treatment ability was additionally determined.

The present retrospective analysis deals with orofacial findings and special orthodontic treatment conditions in patients with trisomy 21 in different stages of the mixed dentition. Treatments according to Castillo-Morales [24] in the first months of age were not considered. Nevertheless, due to the differences in growth this early interdisciplinary treatment concept is very important. In this context, Klingel et al. reported that the palate of DS infants in the first 6 to 9 month of life is of normal shape but considerably smaller compared with healthy normal [30]. From 6 to 9 months onward, the growth pattern of the hard palate in DS infants decreases irregularly. High-arch-constricted palates could, therefore, be interpreted as secondarily acquired in later life. Klingel et al. concluded that it could be advantageous to begin oral muscular stimulating therapy between 6 and 9 months of age which may prevent palatal shape alterations and enhance oral function which also contributes to maxillary development [30]. Also Kaczorowska et al. reported in their systematic review that the use of this plate are very important to ensure better adaptation to the subsequently used apparatus and reduce the risk of disorders of the stomatognathic system [31].

To reduce the subjectiveness and complexity of malocclusion assessment, orthodontic treatment need indices were typically used to rank it [32, 33]. The majority of indices were created to evaluate malocclusion in a specific community or population, making it easier to identify such cases and direct them to treatment [33, 34]. The Dental Aesthetic Index (DAI) [27, 35, 36], the Index of Orthodontic Treatment Needs (IOTN) [8], the Norwegian Need for Orthodontic Treatment Index (NOTI) [37], and the Index of Complexity, Outcome, and Need for Treatment (ICON) [28] have all been used to assess the orthodontic treatment requirements for DS. In Germany, in 2001, an index system was implemented to determine treatment requirements. This system specifies when statutory health insurance funds are obligated to pay for treatment when it is required. Since then, the KIG (Kieferorthopädische Indikationsgruppen) system of orthodontic indication groups must be used by the clinician to assess a patient’s malocclusion severity. The KIG system is close to the IOTN in terms of groups’ form and content; however, the subjective esthetic assessment is not taken into account [21, 38]. To date, the KIG system has not been used to further classify patients with DS in detail, although they are also included in KIG group A5, which means the greatest need for treatment due to the underlying severe malocclusion [21].

In general, the malocclusion of patients with DS is characterized by a Class III tendency and posterior crossbite due to maxillary hypoplasia [1, 16, 39]. With regard to teeth anomalies, a higher incidence of tooth as well as impacted, transposed, and congenitally missing teeth must be expected. Furthermore, tongue thrust and protrusive tongue posture are often associated with an anterior open bite [1].

Concerning an index similar to this used in the present investigation, recently, Alkawari et al. reported in a cross-sectional study using the IOTN index on 23 children aged 10–14 years with DS that approximately 59.1% of them had a class III Angle’s classification, and 47.9% of them had reverse anterior overjet [8]. A posterior crossbite was present in 69.6% of the youngsters, and 13.1% of the children had scissor bites. Further findings were severe crowding in 82.6%, partially erupted teeth in 30.4%, and retained deciduous teeth in 65.2% of the patients. Only 17.4% of the children were found to have a deep overbite. The findings indicated that a higher percentage of the children involved in the research study required treatment (81.9%), with 45.5% of the children categorized within degree 5 of the index (very great need).

Similar initial findings were observed in the present study. With regard to the ANB and WITS, the average deviation from the normal range demonstrated a class III relationship in 75% of all subjects. Considering the transversal differences between the maxilla and mandible, a significant narrowness of the upper dental arch was evident, especially in the posterior segments, which resulted in 60% of all patients in a unilateral (35%) or bilateral (25%) crossbite. Likewise, tooth anomalies regarding congenital hypodontia (85%), tooth impaction (10%), and displacement (15%), as well as apparent hypoplasia, could be confirmed. Tongue dysfunction was also frequently observed, which is probably associated with the tendency of the anterior teeth to be too low and the proclined anterior teeth. Consequently, 65% of all patients received adjuvant logopedic therapy due to caudal tongue position and/or infantile swallowing patterns.

Thus, our study results largely confirm the results already presented in the literature and show that the KIG classification is also a suitable index for describing malocclusion associated with the diagnosis of DS. Due to a large number of severe findings, classification A5 within the KIG classification is also justified, representing the highest need for treatment.

The treatment of patients with DS is basically similar to that of non-syndromic subjects and usually consists of pre-treatment with a removable device followed by a fixed appliance [40–46]. Some studies have proven how orthodontic palatal plate therapy works to treat orofacial dysfunction in children with DS. Bäckman et al. conducted a clinical trial in which children with DS who received orthodontic palatal plates had significantly better oral motor function, facial expression, and speech than those who received no treatment [40]. Carlstedt et al. reported results that were comparable [41]. Recently, Javed et al. evaluated the effects of orthodontic palatal plate therapy in the treatment of orofacial dysfunction in children with DS in a meta-analysis [42]. They found that all studies reported this removable appliance to be effective in improving orofacial disorders in children with DS. Most studies suggest that palatal plate therapy, in combination with language intervention and physiotherapy/orofacial regulation therapy, seems to be effective in improving orofacial disorders in patients with DS. With regard to compliance, the plates were used by 57–65% of the patients without any major problems. However, the use was interrupted mainly because of health problems, and children who exhibited less-than-perfect compliance still used one or both plates during varying time periods. To date, no studies have accounted explicitly for compliance, but the number of dropouts indicates that compliance seems to be significantly lower than in a control group [43, 45, 46].

In contrast to removable appliances, there is little literature available on orthodontic treatment in patients with DS, particularly regarding the use of fixed appliances. Only one study deals with the use of fixed orthodontic devices in patients with DS. Abeleira et al. investigated in a case-control study the use of fixed multibracket dental therapy [47]. They reported that, in patients with DS, orthodontic treatment takes longer than usual and the frequency of complications is higher than in the general population. Furthermore, the authors observed that seven patients (28%) in the DS group required two explanatory desensitization sessions, and even four patients (16%) required three sessions. In addition, only 11 of the 17 patients with DS for whom this was thought to be appropriate received fixed multibracket appliances on both arches [47]. Complications during treatment were more common in the subjects with DS than in the controls due mainly to the appearance of traumatic ulcers, gingival thickening, and deserted oral hygiene. In this context, the mean duration of treatment was 37.48 ± 21.79 months in the patients with DS and 23.56 ± 4.00 months in the control group. Finally, they concluded that orthodontic treatment with fixed appliances in patients with DS is possible, although treatment takes longer and the complication frequency is higher. Therefore, although malocclusions are sometimes severe, the objectives of treatment must be kept simple and realistic [47].

In the present study, the success of treatment was also examined retrospectively, especially concerning patients’ compliance. No differences were made regarding the age at which the treatment was to take place or the type of therapy. It was just proof whether the treatment goal was reached according to the treatment plan. This could be fully, partially, or not achieved. In cases of partial or unsuccessful treatment, the reasons were taken from the patient’s file. It was found that 45% of all patients had to be discontinued during the treatment due to different patient-related reasons. In general, these included insufficient oral hygiene and/or compliance. These criteria for treatment discontinuation essentially apply. However, oral hygiene has a special significance in patients with DS. Even a lower caries risk is reported for this group of patients; in contrast to this, a greatly increased prevalence of developing periodontitis is described [13–15]. In this context, Scalioni et al. confirmed in a systematic review a particular risk for periodontal disease in patients with trisomy 21 [48]. Insufficient cooperation consisted of the repeated loss of removable appliances or their inadequate use, repeated missing appointments, and damage to fixed appliances. In addition, in 25% of the cases, the treatment goal was not achieved due to missing tissue reactions. Overall, this results in an unsatisfactory outcome in 75% of all treatments.

In the course of undesirable courses, the apparently higher occurrence of root resorptions must also be discussed. Of all patients with radiological follow-up, a total of 6 patients (46%) showed isolated to generalized root resorptions. Reasons for idiopathic external apical root resorption are multifaceted [49]. Idiopathic external apical root resorption may be influenced by shortened root development associated with radiation exposure, dentinal dysplasia, and taurodontism [50, 51]. A similar pattern of resorptive defects have been described in combination with various systemic diseases, including hypoparathyroidism, renal disease, and hepatitis [52–54]. In this context, there is very little information in the literature about a possible association with the presence of DS, especially about multiple idiopathic apical root resorption [49, 55]. However, significant reductions in root and crown length and stunted, short, small crowns and roots have been reported in association with DS and are known for a correspondingly higher risk [16, 56]. Nevertheless, a possible association between the determined root resorptions and the presence trisomy 21 must be considered critically, due the incidence of orthodontically induced root resorption vary significantly in the literature and, neither the materials nor the biomechanics were identified the underlying patient records. In this context, the extent of apical root resorptions in context of orthodontic treatment differ considerably in the literature from 0.2 to 2.93 mm [57, 58], and those of radiologically detected root resorptions between 0 and 100% [59].

Due this heterogeneous data set, further research about this topic with larger patient collectives are urgently needed to prove that the observed peculiarities are really related to the gene defect and to figure out, how orthodontic treatment should be designed or adapted that the therapy goal can be achieved even with limited compliance due to disability of the vulnerable patient groups.

## Conclusion

The present results confirm previous orofacial findings in patients with DS. Furthermore, it can be shown that the KIG classification system is excellently suited for a detailed description. Despite a great need for treatment, the treatment is often unsatisfactory due to insufficient compliance and oral hygiene. In addition, the frequent occurrence of root resorption requires further investigation. As a consequence, the treatment plan must be kept simple and realistic to achieve a fast and therapeutically satisfactory treatment result.

## Data Availability

The data supporting the findings of this research can be obtained directly from the corresponding author.
